# Ultrasound-Guided Versus Wire-Guided Breast-Conserving Surgery for Non-palpable Breast Lesions: A Retrospective Review

**DOI:** 10.7759/cureus.72525

**Published:** 2024-10-28

**Authors:** João Mendes, Ana Cláudia Soares, Mariana Peyroteo, Rita Canotilho, Cátia Ribeiro, Joaquim Abreu de Sousa

**Affiliations:** 1 Surgery, Unidade Local de Saúde do Médio Ave, Santo Tirso, PRT; 2 Surgery, Hospital de Santo Espírito da Ilha Terceira, Angra do Heroísmo, PRT; 3 Surgical Oncology, Instituto Português de Oncologia do Porto Francisco Gentil (IPO-Porto), Porto, PRT

**Keywords:** breast cancer, breast-conserving surgery, non-palpable lesions, surgical margins, ultrasound-guided surgery

## Abstract

Background

Breast-conserving surgery (BCS) is standard for early breast cancer, yet achieving clear surgical margins remains challenging. Ultrasound (US)-guided BCS has emerged as a potential alternative to wire-guided surgery, but its efficacy compared to traditional methods requires evaluation.

Methods

A retrospective review of patients undergoing BCS from April 2022 to April 2023 at the Portuguese Institute of Oncology of Porto (IPO-Porto) was conducted. Preoperative assessment by the surgeon determined the choice between ultrasound-guided and wire-guided surgery for non-palpable lesions.

Results

Out of 155 patients, 81 (52.3%) underwent US-guided BCS, while 74 (47.7%) underwent wire-guided BCS. Both groups had similar tumor characteristics and achieved rates of negative surgical margins (69 (92%) versus 53 (93%)). There was no significant difference in intraoperative re-excision rates between the two groups (24 (32%) versus 19 (33.3%); p=8.71). Additionally, the rate of repeat BCS/mastectomy after initial surgery was comparable (6 (8%) versus 4 (7%); p=1.000).

Conclusions

Ultrasound-guided BCS demonstrates comparable efficacy to wire-guided BCS for non-palpable breast lesions. Both techniques provide similar surgical outcomes, with the potential additional benefits of ultrasound-guided BCS for the patient and in the management of healthcare resources.

## Introduction

Breast-conserving surgery (BCS) has established itself as the standard treatment for early-stage breast cancer, with a growing number of procedures being performed due to the widespread implementation of screening programs. This increase in early detection led to a significant reduction in median tumor size [1]. Nevertheless, the incidence of inadequate (close or positive) excision margins ranges from 5% to 60% [2]. Although the optimal margin remains a matter of debate, patients undergo re-excision to obtain clear pathological margins and reduce recurrence rates [3].

Approximately 30% of breast cancers are estimated to be non-palpable at the time of diagnosis [4]. For this kind of lesion, there are various location methods, such as radio-guided or guided by magnetic seed, but wire-guided excision remains the most used method of localization [4]. Ultrasound (US)-guided surgery is suggested as an alternative for the latter [5,6]. It was introduced in the late 80s [7], representing a method in which the surgeon himself can locate the lesion and safely remove it, sparing support and resources from the radiology department. Although several groups have tested the feasibility and oncological safety of this method, results in the current literature remain controversial [8-11].

The aim of the study was to determine and compare the efficacy of US-guided BCS versus the conventional wire-guided BCS for non-palpable breast lesions.

This work was previously presented as an abstract and poster at the 42nd Congress of the European Society of Surgical Oncology, Florence, Italy, October 25-27, 2023 [12].

## Materials and methods

Patient selection

A retrospective, unicentric, comparative study was conducted in patients who underwent BCS from April 2022 to April 2023 at the Breast Clinic Unit of the Portuguese Institute of Oncology of Porto (IPO-Porto), which functions as an integrated multidisciplinary unit within a comprehensive cancer center.

Patients who were referred to our center with nodular lesions that were not clearly palpable on the first appointment were selected for a preoperative consultation, where the surgeon himself performed an ultrasound scan to identify and characterize the previously referred breast lesion.

Those with clearly identified nodules were elected for US-guided surgery, while others with less defined lesions underwent wire-guided surgery. Patients who were undergoing neoadjuvant therapy or had only microcalcifications detected were excluded.

In the wire-guided group, a radiologist from our center used US to place one or more wires on the morning of the surgery. After that, a craniocaudal and mediolateral oblique mammography was done to assess all wire positions.

Surgical excision and margin assessment

In the US-guided group, before incision, a US scan was done by the surgeon in the craniocaudal and transverse axis, measuring the distance from the muscular layer and skin and marking in the skin the excision borders with a sterile marker. After specimen excision, another US scan was done to assess its margins.

Nonetheless, in both groups, during surgery and after excision, the specimen was sent to a pathological extemporaneous examination to assess margins, and if positive, an additional intraoperative excision was done. The specimen weight was assessed by the pathologist and included all intraoperative re-excisions. After surgery, if the margins were positive or insufficient (less than 1 mm on invasive and less than 2 mm on in situ neoplasms), the patients were submitted to a new surgery, after evaluation at the multidisciplinary tumor board.

This study adhered to the principles outlined in the Declaration of Helsinki and was approved by the Ethics Committee of IPO-Porto. Since the statistical analyses were conducted on aggregated data, no individual consent was needed. The raw data, obtained from the health information system, were de-identified to maintain confidentiality, with all personal patient information removed.

Statistical analysis

Descriptive analysis was performed using frequency tables for categorical variables, while continuous variables were summarized using appropriate metrics, such as median and interquartile range (IQR) or mean and standard deviation (SD).

The comparison of categorical variables between the two groups was assessed using either Fisher's exact test or a chi-square test. The means of nonparametric variables were compared using variance analysis. A p-value of less than 0.05 was considered statistically significant. All data analyses were conducted using IBM SPSS Statistics for Windows version 28 (IBM Corp., Armonk, NY).

## Results

Patient and tumor characteristics

After applying the exclusion criteria, a total of 155 patients were enrolled in this study, of which 81 (52.3%) underwent US-guided BCS and the remaining group (n=74 (47.7%)) was submitted to wire-guided BCS. All patients were female. The median age was 59 years old (IQR=16). One-hundred thirty-two (132) patients had malignant lesions (after excluding exclusive ductal carcinoma in situ (DCIS) (n=4) and benign lesions (n=19)), of which 126 (95.5%) were cT1 and 130 (98.5%) were cN0.

When analyzing only the invasive cancer group (n=132), the median lesion size was 11.5 mm (IQR=6), although the US-guided selected lesions appeared to be slightly bigger when compared to wire-guided excision (12 versus 10 mm; p=0.003). Despite this difference, the median size and weight of the surgical specimen did not differ between the two groups (p=0.54 and p=0.495, respectively). The patients and tumor characteristics of this group are summarized in Table 1.

**Table 1:** Patient and tumor characteristics of invasive lesions SD: standard deviation, IQR: interquartile range, US: ultrasound, ER: estrogen receptor, PR: progesterone receptor, HER2: human epidermal growth factor receptor 2

Variable	Wire guided	US guided	t-, U-, or chi-square value	p-value
Number of patients	57	75	-	-
Age, years, mean (SD)	58.6 (9.2)	60.7 (12.5)	-1.669	0.098
Tumor size, mm, median (IQR)	10 (7)	12 (5)	1505	0.003
Tumor weight, g, median (IQR)	38.2 (36.3)	43.8 (39.9)	1989	0.495
Menopausal status, number (%)	-	-	2.031	0.154
Premenopausal	21 (15.9)	19 (14.4)	-	-
Postmenopausal	36 (27.3)	56 (42.4)	-	-
Histological type, number (%)	-	-	5.074	0.079
Ductal	41 (31.1)	65 (49.2)	-	-
Lobular	12 (9.1)	6 (4.5)	-	-
Other	4 (3)	4 (3)	-	-
Histological grade, number (%)	-	-	3.283	0.194
Grade 1	3 (2.3)	4 (3.1)	-	-
Grade 2	43 (33.3)	44 (34.1)	-	-
Grade 3	11 (8.5)	24 (18.6)	-	-
ER status, number (%)	-	-	0.021	1.000
Positive	55 (41.7)	72 (54.5)	-	-
Negative	2 (1.5)	3 (2.3)	-	-
PR status, number (%)	-	-	2.016	0.156
Positive	52 (39.4)	62 (47)	-	-
Negative	5 (3.8)	13 (9.8)	-	-
HER2 status, number (%)	-	-	1.055	0.304
Positive	4 (3.1)	9 (7)	-	-
Negative	53 (41.1)	63 (48.8)	-	-
Ki67 status, number (%)	-	-	8.978	0.030
<15%	22 (22.9)	18 (18.8)	-	-
15%-30%	13 (13.5)	21 (21.9)	-	-
>30%	5 (5.2)	7 (7.3)	-	-

Tumor margins

Intraoperatively, all lesions were identified by ultrasound. After intraoperative examination, 43 (32.6%) patients required intraoperative margin re-excision, and no difference between re-excision rate was found between the two groups (24 (32%) in the US-guided versus 19 (33.3%) in the wire-guided group; p=0.871), as described in Table 2.

**Table 2:** Re-excision rates according to localization method US: ultrasound

Variable	Wire guided	US guided	Chi-square value	p-value
Total	57	75	-	-
Intraoperative re-excision, number (%)	19 (33.3)	24 (32)	0.026	0.871
Second operation	4 (7)	6 (8)	0.045	1.000

Considering the final pathology examination, most of the tumors were excised with adequate margins (n=122; 92.4%). In the US-guided BCS group, the adequate margin rate was obtained in 69 (92%), comparable to the wire-guided group (n=53; 93%).

A 7.6% (n=10) rate of repeat BCS/mastectomy after first surgery was obtained, and this rate was similar between the two groups (6 (8%) in the US-guided group versus 4 (7%) in the wire-guided group; p=1.000).

When considering tumor characteristics, no influence on margin status was found regarding age, tumor size, tumor weight, histological type, menopausal status, estrogen and progesterone receptor status, and Ki67 status. In contrast, an association between insufficient margins and positive HER2 status (p=0.0047) and high histological grade (p=0.002) was found. The intraoperative localization method also did not have an effect on margin status. The influence on surgical margins regarding patients and tumor characteristics is described in Table 3.

**Table 3:** Margin status according to patients and tumor characteristics SD: standard deviation, IQR: interquartile range, ER: estrogen receptor, PR: progesterone receptor, HER2: human epidermal growth factor receptor 2

Variable	Negative	Positive	t-, U-, or chi-square value	p-value
Total (%)	122 (92.4)	10 (7.6)	-	-
Age, years, mean (SD)	60.5 (9.7)	57.5 (12.7)	0.908	0.210
Tumor size, mm, median (IQR)	11 (6)	12.5 (5)	433.5	0.127
Tumor weight, g, median (IQR)	40.6 (38.3)	54.1 (37.1)	516.5	0.421
Localization method, number (%)	-	-	0.045	1.000
Wire guided	53 (93)	4 (7)	-	-
US guided	69 (92)	6 (8)	-	-
Menopausal status	-	-	0.482	0.490
Premenopausal	36 (90)	5 (10)	-	-
Postmenopausal	86 (93.5)	6 (6.5)	-	-
Histological type	-	-	4.944	0.084
Ductal	98 (92.5)	8 (7.5)	-	-
Lobular	18 (100)	0 (0)	-	-
Other	6 (75)	2 (25)	-	-
Histological grade	-	-	12.61	0.002
Grade 1	7 (100)	0 (0)	-	-
Grade 2	85 (97.7)	2 (2.3)	-	-
Grade 3	28 (80)	7 (20)	-	-
ER status	-	-	1.146	0.330
Positive	118 (92.9)	9 (7.1)	-	-
Negative	4 (80)	1 (20)	-	-
PR status	-	-	0.121	1.000
Positive	105 (92.1)	9 (7.9)	-	-
Negative	17 (94.4)	1 (5.6)	-	-
HER2 status	-	-	5.774	0.047
Positive	10 (76.9)	3 (23.1)	-	-
Negative	110 (94.8)	6 (5.2)	-	-
Ki67 status	-	-	5.617	0.132
<15%	39 (97.5)	1 (2.5)	-	-
15%-30%	33 (97.1)	1 (2.9)	-	-
>30%	11 (91.7)	1 (8.3)	-	-

## Discussion

BCS, combined with adjuvant chemoradiotherapy, emerged as the new standard of care for early breast cancer, with the same effectiveness and oncological safety as mastectomy [13]. However, the challenge behind this paradigm shift remains the attainment of correct surgical margins. Local recurrence is directly associated with poor resection margins [3], resulting in more morbidity and costs associated with a new intervention for a re-excision [14,15]. This attests to the crucial importance of obtaining intraoperative clear surgical margins in BCS.

This study aimed to determine and compare the efficacy of our institutional standard practice of wire-guided BCS versus US-guided BCS on non-palpable breast lesions. Our findings suggest that ultrasound-guided BCS is as effective as wire-guided BCS in achieving clear surgical margins and minimizing re-excision rates, without compromising safety and feasibility.

Ultrasound serves as a key tool for guiding breast biopsies and diagnostic interventions [5,6]. US-guided BCS is recognized as a promising technique for achieving clear surgical margins [16-18] with recognized feasibility and security [19,20]. In our study, it was used not only during surgery but also preoperatively by the surgeon to assess lesion characteristics and BCS surgery feasibility with clear surgical margins. This enabled a more rigorous selection of patients who benefit from ultrasound-guided surgery, as it allows preoperative planning of the surgical approach, thus conserving wire usage for patients truly in need.

The majority of the studies found in the literature comparing wire- and US-guided BCS surgery describe only a small series, and we could only identify one prospective randomized paper published regarding this subject [9]. Our study analyzed 132 patients in a one-year period, with well-balanced arms (57 (43.2%) in the wire-guided group and 75 (56.8%) in the US-guided group) with no differences between groups regarding preoperative characteristics, despite slightly larger lesions in the ultrasound group. No differences were found between groups regarding surgical margin status and intraoperative and second re-excision rates, which demonstrates the non-inferiority of using US in BCS. One of our main concerns was that a bigger volume of breast tissue would be removed in US-guided surgery. However, when comparing the size and weight of the surgical specimen, a significant difference was also not found between groups.

Eggemann et al. [8] published a series with 158 patients in a six-year span and not only found no disadvantage comparing US-guided to wire-guided excision but also obtained a significant intraoperative re-resection reduction in the US group. Hu et al. [9] published a prospective randomized study with 520 patients submitted to surgery between June 2010 and January 2015 and stated that US-guided BCS leads to reduced re-excision rates and better margin clearance. Our results varied, primarily because we were in the early stages of the learning curve, and so, the use of ultrasound was focused primarily on intraoperative lesion detection instead of margin assessment. It should also be reinforced that all lesions in our series, regardless of the group, were sent to an extemporaneous examination and assessed by a dedicated pathologist.

In fact, besides localization, one of the possible advantages of intraoperative US lies in its ability to assess margins during lesion extraction, complementing palpation-guided methods. Our work stated that the intraoperative re-excision rate did not differ significantly between wire-guided and US-guided groups (19 (33.3%) versus 24 (32%); p=0.871), demonstrating that US is not superior but is as safe as using wire.

In Table 2, we described possible factors to predict positive margins. Our study concluded that neither wire-guided nor US-guided surgery led to more positive margins (p=1.00), but we found a relationship between positive margins and histological grade (p=0.002) and HER2 status (p=0.047), maybe due to the more aggressive behavior of high histological grade and HER2-positive lesions [20,21].

There are additional benefits linked to the utilization of ultrasound (US) in this scenario. From the patient's perspective, this technique provides increased comfort, reduced trauma and pain, and fewer hospital visits for wire insertion [22-25]. Moreover, it is associated with cost savings and can alleviate the workload of radiologists and pathologists [26,27]. From the surgeon's standpoint, it places the decision-making process in their hands, allowing for intraoperative adjustments, although requiring proficiency in US and its associated learning curve.

The retrospective nature of this study constitutes one of its limitations. The satisfaction of the patient was not evaluated, and the groups were not randomized but based on the surgeon's decision to propose US-guided surgery. As strong points, we have a high proportion of patients in only a one-year span, reducing data bias, and a similar proportion between groups, decreasing selection bias.

## Conclusions

In conclusion, our study demonstrates that for non-palpable breast lesions, ultrasound-guided and wire-guided breast-conserving surgery (BCS) have comparable efficacy, with both techniques achieving similar surgical outcomes. The use of ultrasound not only ensures precise lesion localization but also spares valuable radiology resources, making it a practical and efficient tool in clinical practice. Furthermore, the patient-centered benefits, including reduced discomfort and fewer preoperative procedures, suggest that US-guided BCS could become a preferred method in managing non-palpable breast lesions, particularly in high-volume cancer centers where resource optimization is critical.

## References

[1] Lovrics PJ, Cornacchi SD, Farrokhyar F, Garnett A, Chen V, Franic S, Simunovic M (2009). The relationship between surgical factors and margin status after breast-conservation surgery for early stage breast cancer. Am J Surg.

[2] Eggemann H, Ignatov T, Beni A, Costa SD, Ignatov A (2014). Ultrasonography-guided breast-conserving surgery is superior to palpation-guided surgery for palpable breast cancer. Clin Breast Cancer.

[3] Luini A, Rososchansky J, Gatti G (2009). The surgical margin status after breast-conserving surgery: discussion of an open issue. Breast Cancer Res Treat.

[4] Micha AE, Sinnett V, Downey K (2021). Patient and clinician satisfaction and clinical outcomes of Magseed compared with wire-guided localisation for impalpable breast lesions. Breast Cancer.

[5] Fornage BD, Sneige N, Edeiken BS (2002). Interventional breast sonography. Eur J Radiol.

[6] Nurko J, Edwards MJ (2005). Image-guided breast surgery. Am J Surg.

[7] Schwartz GF, Goldberg BB, Rifkin MD, D'Orazio SE (1988). Ultrasonography: an alternative to x-ray-guided needle localization of nonpalpable breast masses. Surgery.

[8] Eggemann H, Costa SD, Ignatov A (2016). Ultrasound-guided versus wire-guided breast-conserving surgery for nonpalpable breast cancer. Clin Breast Cancer.

[9] Hu X, Li S, Jiang Y, Wei W, Ji Y, Li Q, Jiang Z (2020). Intraoperative ultrasound-guided lumpectomy versus wire-guided excision for nonpalpable breast cancer. J Int Med Res.

[10] Vartanian A, Papas PV, Guarecuco Castillo JE, Sistare M, Masri MM (2023). Ultrasound guided intraoperative wire localization under general anesthesia in breast-conserving surgery. Cureus.

[11] Fosko NK, Gribkova Y, Krupa K (2023). The use of intraoperative ultrasound during breast conserving surgery. Clin Breast Cancer.

[12] Matos Mendes JF, Peyroteo M, Canotilho R (2024). Ultrasound-guided versus wire-guided breast-conserving surgery for non-palpable breast lesions: retrospective review. Eur J Surg Oncol.

[13] Fisher B, Anderson S, Bryant J (2002). Twenty-year follow-up of a randomized trial comparing total mastectomy, lumpectomy, and lumpectomy plus irradiation for the treatment of invasive breast cancer. N Engl J Med.

[14] Kaufmann M, Morrow M, von Minckwitz G, Harris JR (2010). Locoregional treatment of primary breast cancer: consensus recommendations from an international expert panel. Cancer.

[15] Morrow M (2010). Trends in the surgical treatment of breast cancer. Breast J.

[16] Vispute T, Seenu V, Parshad R, Hari S, Thulkar S, Mathur S (2018). Comparison of resection margins and cosmetic outcome following intraoperative ultrasound-guided excision versus conventional palpation-guided breast conservation surgery in breast cancer: a randomized controlled trial. Indian J Cancer.

[17] Hoffmann J, Marx M, Hengstmann A (2019). Ultrasound-assisted tumor surgery in breast cancer - a prospective, randomized, single-center study (MAC 001). Ultraschall Med.

[18] Arko D, Čas Sikošek N, Kozar N, Sobočan M, Takač I (2017). The value of ultrasound-guided surgery for breast cancer. Eur J Obstet Gynecol Reprod Biol.

[19] Ahmed M, Douek M (2013). Intra-operative ultrasound versus wire-guided localization in the surgical management of non-palpable breast cancers: systematic review and meta-analysis. Breast Cancer Res Treat.

[20] Asif HM, Sultana S, Ahmed S, Akhtar N, Tariq M (2016). HER-2 positive breast cancer - a mini-review. Asian Pac J Cancer Prev.

[21] Rakha EA, Reis-Filho JS, Baehner F (2010). Breast cancer prognostic classification in the molecular era: the role of histological grade. Breast Cancer Res.

[22] Pan H, Wu N, Ding H (2013). Intraoperative ultrasound guidance is associated with clear lumpectomy margins for breast cancer: a systematic review and meta-analysis. PLoS One.

[23] Donaldson LA, Cliff A, Gardiner L, Hubbard AE, Ashton MA (2003). Surgeon-controlled ultrasound-guided core biopsies in the breast--a prospective study and a new use for surgeons in the clinic. Eur J Surg Oncol.

[24] Krekel NM, Lopes Cardozo AM, Muller S, Bergers E, Meijer S, van den Tol MP (2011). Optimising surgical accuracy in palpable breast cancer with intra-operative breast ultrasound--feasibility and surgeons' learning curve. Eur J Surg Oncol.

[25] Staren ED, Knudson MM, Rozycki GS, Harness JK, Wherry DC, Shackford SR (2006). An evaluation of the American College of Surgeons' ultrasound education program. Am J Surg.

[26] James TA, Harlow S, Sheehey-Jones J (2009). Intraoperative ultrasound versus mammographic needle localization for ductal carcinoma in situ. Ann Surg Oncol.

[27] Haloua MH, Krekel NM, Coupé VM, Bosmans JE, Lopes Cardozo AM, Meijer S, van den Tol MP (2013). Ultrasound-guided surgery for palpable breast cancer is cost-saving: results of a cost-benefit analysis. Breast.

